# Is There a Time and a Place for the Gluten-Free Diet in Potential Celiac Disease?

**DOI:** 10.3390/nu15184064

**Published:** 2023-09-20

**Authors:** Roxana Nemteanu, Andreea Clim, Corina Elena Hincu, Liliana Gheorghe, Irina Ciortescu, Anca Trifan, Alina Plesa

**Affiliations:** 1 Medical I Department, “Grigore T. Popa” University of Medicine and Pharmacy, 700115 Iasi, Romania; 2 Institute of Gastroenterology and Hepatology, “Sfantul Spiridon” University Hospital, 700111 Iasi, Romania; 3 Department of Radiology, “Sfantul Spiridon” University Hospital, 700111 Iasi, Romania

**Keywords:** potential celiac disease, gluten-free diet, risk factors

## Abstract

Potential celiac disease (PCD) is characterized by the absence of villous atrophy on duodenal biopsies (Marsh 0 or 1) despite positive celiac serology and HLA DQ2 or DQ8 heterodimers. Recent epidemiological studies report that PCD represents one fifth of the total CD diagnoses. Compared to patients with CD, the majority of adult patients with PCD show lower rates of nutrient deficiencies and extraintestinal symptoms at diagnosis. Recommending a gluten-free diet (GFD) to PCD patients depends on whether they have symptoms or not. A significant clinical improvement is reported by symptomatic patients, but for asymptomatic PCD, diet implementation is still a matter of debate. Some questions remain to be answered: does PCD serve as an intermediary phase leading to the progression of true CD? Is it reasonable to hypothesize that PCD and active CD represent different manifestations of the same condition? Is there a potential for both underdiagnosis and overdiagnosis of CD in those who may have the condition? Additional research is required to address these inquiries and ascertain the specific subset of people with potential progression to overt CD, as well as to determine the potential advantages of early implementation of a GFD for these individuals. The investigation of risk factors in CD warrants examination of variables such as the timing of diagnosis, the genetic profile, the extent of gluten exposure, and the composition of the microbiome.

## 1. Introduction

Celiac disease (CD) is a systemic autoimmune disorder caused by gluten consumption [1]. The celiac population is a multivariate heterogeneous cluster of individuals with different genetic and clinical profiles [2]. At present, the global prevalence of CD stands at 1.4%, with an annual growth rate of approximately 7.5%. This escalating incidence underscores the significance of CD as a pressing health concern [3]. The impact of gluten consumption on the rising incidence rates remains uncertain. However, it can be asserted that improved screening and diagnostic procedures, facilitated by the widespread availability of accurate tests and increased awareness among general practitioners and other medical professionals, have contributed to a more effective identification and diagnosis process [4]. However, a substantial proportion of patients remain undiagnosed and the reasons include subtle symptoms, inappropriate interpretation of serologic testing, and inadequate interpretation and recovery of duodenal biopsies. Confirmation of a CD diagnosis is established based on a combination of signs and symptoms, positive specific serology, villous atrophy (VA), genetic factors, and a positive response to the gluten-free diet (GFD). However, the range of signs and symptoms caused by gluten is extensive, ranging from classical CD to more challenging subtypes that are more difficult to detect, such as potential CD (PCD), silent CD, or subclinical CD [5]. According to recent epidemiological studies, it has been found that PCD accounts for 20% of all diagnosed cases of CD [6]. Based on the Oslo definitions, the diagnosis of PCD is characterized by the concurrent presence of distinct celiac autoimmune markers, namely anti-tissue transglutaminase IgA (tTG2 IgA) and anti-endomysium IgA (EMA IgA), in addition to testing positive for HLA-DQ2 and/or -DQ8, and exhibiting non-atrophic mucosal alterations [7]. Patients with PCD may be symptomatic or asymptomatic, having intestinal or extra-intestinal manifestations. Isolated low antibody levels should be interpreted cautiously as autoimmune diseases, chronic liver disease, and infections may be associated with a positive test [8]. Some questions remain to be answered: does PCD serve as an intermediary phase leading to the occurrence of true CD? Is it reasonable to hypothesize that PCD and active CD represent different manifestations of the same pathological condition? Is there a potential for both underdiagnosis and overdiagnosis of CD in those who may have the condition? [9]. Additional research is required to address these inquiries and ascertain the specific subset of people with potential progression to overt CD, as well as to determine the potential advantages of early implementation of a GFD for these individuals [10]. The investigation of risk factors in CD warrants examination of variables such as the timing of diagnosis, the genetic profile, the extent of gluten exposure, and the composition of the microbiome.

### 1.1. Is PCD on the Road toward True Celiac Disease?

The natural course of CD is not as clearly understood as one might assume. CD has been characterized as an iceberg disease, and the celiac iceberg refers to diagnosing based on more obvious signs, symptoms, or biochemical abnormalities [10,11,12]. PCD is the hidden submerged part of the iceberg. “True” PCD is a discrete entity somewhere on the CD spectrum, with a combination of gluten-induced symptoms, mild or no enteropathy, positive serology, and genetic susceptibility. It is argued that the onset of PCD and possible progression toward overt CD are influenced by the genetic makeup [11,12]. Patients having PCD have been shown to possess a low-to-moderate HLA-related risk with a higher prevalence of the DQ8 genotype or genes encoding half of the DQ2 heterodimer [13]. A “gene-dosage” effect has been reported for HLA and could offer a partial explanation for the onset of the disease. The clinical diagnosis of PCD can be complex, and the clinical significance of PCD remains unclear [10,13]. Establishing a certain diagnosis of PCD may therefore be challenging.

The histological changes in PCD occur in the small intestine. The mucosal lesions are unevenly distributed and patchy [14]. Currently, the Marsh–Oberhuber classification is recommended by different gastroenterology and pathology societies [15]. It is used as a semiquantitative system offering a subjective description of IELs, crypt hyperplasia, and VA changes. As such, current guidelines encourage taking numerous biopsy samples from both the bulb (1–2 specimens) and duodenum (4–6 specimens) to allow recovery of well-oriented specimens and to reduce the number of false negative samples [16,17]. Limited tissue sampling and poor orientation are major preanalytical factors impacting proper histological evaluation. It is also recommended to have only one biopsy per pass during standard endoscopy to ensure tissue integrity [17,18]. As previously reported by different publications, the histological lesions identified in the duodenum are not pathognomonic for CD, and other etiologies should be suspected. In PCD, mild enteropathy expressed by increased intraepithelial lymphocyte count can be easily mistaken for other conditions. For example, Helicobacter pylori gastritis is associated with an increased count of IELs in the duodenal mucosa mimicking CD [19]. More recently, in addition to Olmesartan, an angiotensin 2 receptor blocker, immune checkpoint inhibitors have been linked to duodenal changes encountered in CD [20]. It is, however, very difficult to differentiate CD from mimickers in clinical practice, especially when antibody levels are extremely low or normal [21].

However, CD-specific autoimmunity EMA and tTG2 IgA antibody levels are persistently increased in PCD patients [19]. EMA is more specific than tTG2 because an isolated positivity for tTG2 at low titer can also be found in conditions other than CD [22]. Analytical or random errors in the assay of antibody levels can occur; therefore, a surveillance protocol should be implemented in such patients. When assessing the prediction of progression toward overt CD, the most important aspect was the persistence of antibody positivity of EMA and tTG2 during follow-up [21]. A single positive value for EMA or tTG2 is not sufficient for a positive PCD diagnosis. It has been argued that circulating tTG2 antibodies could be a consequence of an overabundance of the tTG2 deposits from the mucosal intestinal layer [23,24,25,26]. Early discovery of tTG2 deposits may offer insight into the possible development of early-stage overt CD. Tosco et al. discovered that these deposits share the same patchy distribution alternating with areas without tTG2 deposits as seen in active CD, and isolated bulb involvement is not exceptional, supporting guideline requirements for multiple biopsy sampling [27]. 

### 1.2. Risk Factors for Progression from Mild to Villous Atrophy in CD

**Figure 1:**
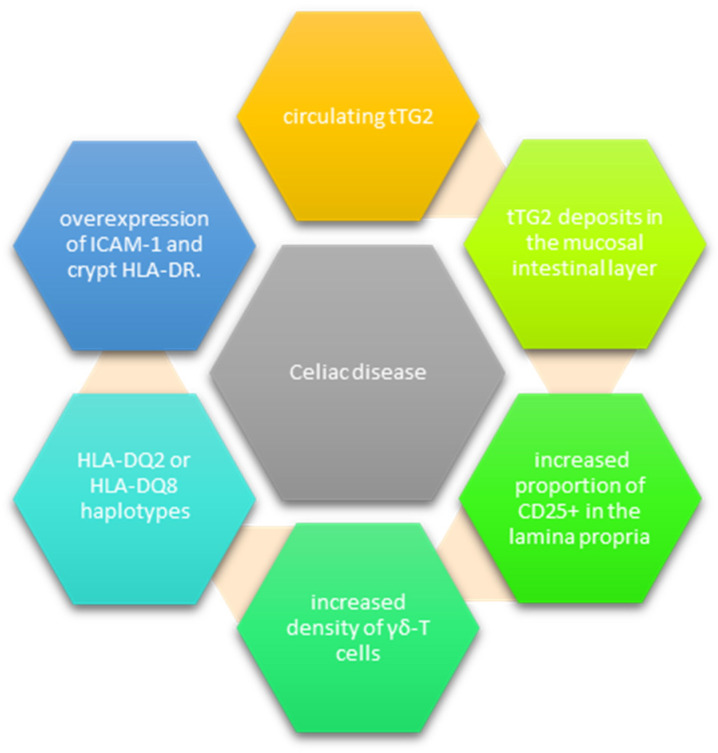
Markers of existing early celiac disease.

Even though PCD patients do not experience significant mucosal injury, clear signs of inflammation are often identified [21]. Current research has shown that the prognostic factors for VA are represented by the tTG2 deposits in the duodenal mucosa, the increased proportion of CD25+ in the lamina propria and the increased density of γδ-T cells, and the overexpression of ICAM-1 and crypt HLA-DR [28] (Figure 1). Intestinal tTG2 IgA antibody deposits, although seldom reported in PCD, offer an important insight into disease progression [25,26,27]. They are usually identified below the basement membrane, along the villous and crypt, and around the vessels and can be detected before antibodies pass into circulation. In addition, detecting these deposits and negative serology could help identify patients with gluten sensitivity [21,27].

The detection of tTG2 deposits was correlated with an increased risk for progression toward VA, as reported by Salmi et al. Cohort studies performed in at-risk children, who were screened for CD autoimmunity, have shown that a significant percentage have PCD [29]. In addition, Tosco et al. reported in a cohort of 106 children with PCD that after 3 years of a gluten-containing diet, one-third developed VA, while the majority remained healthy. Intestinal deposits of tTG2 identify children at risk for VA [30]. Similar results were reported by Sakhuja et al. in a small retrospective study where 4% of children who initially had a negative biopsy later developed VA [31]. Lionetti et al. reported in a recent study the results from the follow-up CELIPREV study that assessed children with CD-predisposing HLA genotypes from birth. The patients continued a normal gluten-containing diet and were assessed serologically every year. A biopsy was performed on patients with persistent positive serology. Overall, 4.7% were classified as PCD and continued a normal diet. After 10 years of follow-up, only three (13%) children developed overt CD, confirming that the risk of progression toward typical CD is very low. The sole significant predictor for the development of overt CD in the pediatric population was the persistent positive value of tTG2 and EMA during follow-up [32]. Nonetheless, Auricchio et al. reported a cumulative incidence of progression to VA of 43% at 12 years for a cohort of 280 Italian children. In the context of multivariate analysis, the baseline parameters that exhibited the strongest association with the development of VA were the quantities of γδ IELy cells, followed by age and homozygosity for the HLA DQB1*02 allele [33].

Previous investigations have documented the occurrence of loss or variation in serum CD autoimmunity. However, the underlying cause for the spontaneous disappearance of CD autoantibodies in individuals with PCD remains uncertain [33]. In a study by Simell et al., despite continuing gluten exposure, positive tTG and EMA levels were spontaneously lost in almost half of the children. CD antibody loss or fluctuation was not associated with the potential form of CD [34]. Järvinen et al. conducted a study to assess whether the density of small-intestinal villous tip IELy would be of value in clinical practice in uncovering early-stage CD. Several parameters such as the villous tip CD3+ and γδ+ IELy were assessed in the three groups of patients: definite early-stage CD without VA, classic CD, and controls. The authors reported a higher number of villous tip IELs in patients diagnosed with early-stage CD compared to controls [35]. However, the increased number of γδ+ IELy is not pathognomonic for CD, as suggested by other authors. Borelli et al. reported that children with PCD exhibit lower levels and lower density in the lamina propria of IL-21 and IL-17a when compared with active CD. In PCD, IL-21 production, but not IL-17a, can be induced by gliadin and stimulated by IL-15. The role of IL-21 as a regulatory cytokine of the innate and adaptive immune responses can be speculated as this molecule has a major influence on the occurrence of VA [36].

Previous work conducted by Granito et al. addressed an important and underrated serological marker that should be kept in mind when aiming to detect progression toward VA of PCD patients. The authors monitored the behavior of antimicrofilaments IgA both before and after gluten withdrawal. Interestingly, the disappearance of antimicrofilaments IgA was observed in the 20 patients who underwent testing, following the elimination of gluten, in line with the process of histological healing [37]. The present investigation by Granito et al. demonstrates a noteworthy association between antimicrofilament IgA and the extent of intestinal injury in individuals with untreated CD. The cessation of antimicrofilament IgA following the elimination of gluten is indicative of the restoration of the intestinal mucosa and may be regarded as a valuable tool for monitoring individuals with both active CD and PCD. While antimicrofilament IgA antibodies have limited sensitivity and cannot replace EMA and anti-tTG2 IgA in the diagnostic algorithm for CD, their presence is specifically associated with flat mucosa and indicates a severe gluten-sensitive enteropathy [37]. Additionally, they may serve as a complementary tool for monitoring severe CD, alongside traditional markers. Their role could be extended toward monitoring PCD patients with mild or absent enteropathy, but future studies are required to test this hypothesis.

One potential rationale for the existence of a “gray area” encompassing several patients exhibiting indications of autoimmunity related to CD, while missing the characteristic CD enteropathy, may be attributed to genetic factors. CD is predominantly characterized as an immunological condition that is mediated by T cells, specifically CD4+ T cells, and involves the significant involvement of HLA class II molecules. The presence of an innate immune response to gluten is necessary for the progression of VA, as indicated by previous research [34]. The progressive stages could be explained at least partially by the genetic background. An explanation for the absence of VA could be the lack of innate activation of epithelial cells, and a lower level of proinflammatory adaptive anti-gluten immunity might also be responsible [36]. The examination of candidate genes linked with CD that are not related to the HLA revealed a distinct gene pattern in patients with PCD. This suggests that the genetic makeup of individuals may influence the clinical manifestation of CD in a unique manner [38].

Sperandeo et al. researched the genetic profile trying to clarify why PCD has the celiac HLA haplotypes and positive antibodies but do not develop mucosal injury [38]. Interestingly, the authors identified two PCD populations with different non-HLA genes and different expressions of other candidate genes. The normal mucosa M0 in PCD patients exhibited an abundance of IL-2 and KIAA1109 compared to M1, CD, and control patients [10].

Upadhyay et al. proposed a different outlook for early detection of progression from PCD to CD, suggesting the metabolomics approach using resolution nuclear magnetic resonance. Specifically, the authors hypothesized that understanding the metabolic profile of patients with PCD (intestinal, blood, and urinary metabolome) may provide knowledge of the early biochemical changes associated with CD pathogenesis. Interestingly, the authors assessed the metabolic profile of intestinal mucosal biopsies and identified 29 metabolites in both CD and PCD patients. PCD patients reported significantly higher levels of glycerophosphocholine (GPC) and lower levels of allantoin, tyrosine, and tryptophan compared to CD patients [39]. The presence of GPC, a membrane metabolite that is used for the renewal of enterocytes, is indicative of the active renewal of enterocytes while maintaining a normal villous structure. Lower levels of tryptophan and its metabolite indole-3-propionic acid (which confers protection against oxidative damage) suggest an effective use of this metabolite, thus preserving a healthy mucosa [39,40]. 

Furthermore, in cases where there are changes in the quantities of specific amino acids (such as histamine, glycine, and arginine), it is anticipated that there would be a decrease in the protective activity of cells, resulting in an elevated susceptibility of patients to the gradual development of inflammation in the mucosal lining. The researchers reached the conclusion that patients with PCD exhibit a distinct metabolic profile in their intestinal mucosa, blood plasma, and urine [39]. 

In the intestinal mucosa, the metabolites histidine, glycine, tyrosine, tryptophan, and isoleucine have shown the ability to differentiate between individuals with PCD and those without the condition. Comparable findings were observed upon assessing the levels of six metabolites in the bloodstream of individuals with PCD compared to healthy controls. The patients diagnosed with PCD exhibited reduced levels of oxidative stress, normal membrane metabolism, and distinct gut bacteria in comparison to individuals with CD. These factors may potentially account for the observed maintenance of normal mucosal integrity in PCD [39,41].

### 1.3. Treatment for PCD: Who to Treat? When to Treat? For How Long?

Gluten consumption is required for the production of antibodies against gliadin and the self-antigen tTG2 [42]. Antibody levels against tTG2 decrease substantially when reducing or eliminating dietary gluten. Seroconversion is to be expected in patients adhering to a GFD, which usually occurs in a few weeks. In general, the term “gluten-free” describes a level of gluten that is regarded as safe for people with CD. Currently, a level below 20 parts per million of gluten is considered safe. The severity of mucosal injury seems to be correlated with the presence of a celiac-specific serology at diagnosis [2,11].

However, PCD patients experience minor or no histological changes, and, as expected, lower antibody levels. In the recent paper by Newton et al., when assessing the tTG2 levels, the authors found lower levels in PCD patients compared to their atrophic CD counterparts [43]. There are sufficient data to assert that higher tTG2 titers are predictive of VA [44]. Therefore, clinicians will associate lower antibody levels with mild enteropathy on biopsy samples. It is also very important to establish gluten intake around the time of endoscopy; in “true” PCD, the individual will be adequately exposed to gluten and should not be mistaken for those who have partial mucosal recovery through the reduction in gluten [45].

Nevertheless, it is important to evaluate serum EMA or tTG2 IgA antibodies in individuals who exhibit an unexplained elevation in liver enzymes, have a history of atopy, suffer from micronutrient deficiencies, or experience anemia. This recommendation applies to both pediatric and adult populations. The progression of anemia and iron deficiency in individuals with CD occurs along a spectrum and can manifest even in children with intact villous morphology [46,47]. This underscores the importance of promptly identifying and potentially managing these patients through early diagnosis and dietary interventions [47]. As previously reported, not all celiacs are equal and CD should be actively screened and categorized among at-risk populations.

After the diagnosis of CD, implementation of a life-long GFD is required [48]. The GFD has withstood the test of time and has proven its efficacy in small bowel recovery while improving the signs and symptoms of malabsorption and the quality of life (QoL) for CD patients, as well as reducing the overall disease burden [49]. However, for PCD patients who do not share the same morphological abnormalities as typical CD, recommending a GFD is still a matter of debate. Recent studies have indicated that adherence to a GFD can significantly impact an individual’s quality of life (QoL) [50]. Patients who are at risk to develop VA may lose their positive celiac antibodies or continue to have positive serologic tests without developing VA [6,10,11]. Hence, the accurate identification of overt CD or PCD and the determination of the appropriate treatment necessitate a high level of precision.

These serologically positive cases in the absence of symptoms are more often monitored and not treated with a GFD. However, when symptoms are present, a GFD may benefit patients. In PCD patients, the development of adaptive anti-gluten immunity is not sufficient to develop VA [2,51]. Therefore, a GFD would outweigh the benefits as it cannot be used for preventive purposes. Some risk factors have already been discussed that could predict progression toward CD, which can be kept in mind when a decision has to be made. For example, Kurppa et al. conducted a study to examine the impact of a GFD on individuals with asymptomatic CD and PCD. The findings of their research indicated that the introduction of a GFD resulted in poorer social experiences for the participants. The researchers concluded that individuals who exhibit no obvious symptoms but test positive for antibodies would benefit from a GFD [52].

Mandile et al. reported the results of a prospective study involving 65 children with PCD treated with the GFD. The authors concluded that after one year of treatment, only half of the patients showed a significant clinical improvement. However, no differences were observed in terms of Marsh grading, IELy density, and tTG2 deposits [53]. In a large follow-up cohort study of PCD patients, the authors confirmed that 70.7% of patients showed clinical improvement. The patients who did not gain a clinical benefit from the GFD proved to have unrelated causes of their symptoms such as irritable bowel syndrome or microscopic colitis [43]. However, reports by Lionetti et al. and Tosco et al. argue against recommending the GFD in symptomless PCD patients [23,30]. This adds to the uncertainty of recommending the GFD in PCD patients because, while there may be a justifiable use for the GFD in symptomatic PCD, in situations where a patient has minimal or a lack of symptoms, is the adoption of an onerous GFD with regular follow-up necessary? Table 1 contains some of the studies involving PCD patients and the outcome of implementation of a GFD vs. no diet. Recent guidelines recommend strict adherence to the GFD, surveillance by a specialist and/or a dietician to monitor disease and metabolic complications, and regular follow-up. The endpoint of these endeavors is to obtain mucosal healing. For PCD patients who show mild or no histological damage, implementation of the GFD remains controversial [2,4,7].

In conclusion, CD is a common disease with different genetic and phenotypic profiles. PCD can be considered a precursor of true overt CD because we can identify similar genetic backgrounds and antibodies with variable potential for progressing toward VA. In essence, all individuals who advanced to VA maintained their gluten consumption, but not all individuals who persisted in consuming gluten advanced to VA. This prompts a thought-provoking discussion regarding the necessity of a GFD in patients with PCD. While the GFD is undoubtedly beneficial for managing symptomatic PCD, it is worth considering whether it is essential to implement a burdensome GFD with regular monitoring for patients who exhibit minor or no symptoms. Based on the data presented, it appears most suitable to elucidate the concept of ambiguity to those diagnosed with asymptomatic PCD, while concurrently providing assistance and guidance to those who opt to maintain gluten exposure, through consistent and periodic monitoring.

## Figures and Tables

**Table 1:** Summary of results from available data concerning implementation of GFD in PCD patients.

Study	Year	Type of Study and Timeline	Study Population	Gluten-Free Diet Treatment Yes/No	Closing Remarks
Kurppa et al. [54]	2009	randomized, controlled, clinical trial from 2003 to 2008	23 adults	yes	Patients with EMA benefit from a GFD regardless of the degree of enteropathy. All participants chose to continue on a life-long GFD.
Kurppa et al. [55]	2010	Case–control clinical trial	17 pediatric patients	yes	The research indicated that, despite exhibiting a normal duodenal structure, children who test positive for EMA antibodies have a celiac-type condition and could benefit from early treatment.
Tosco et al. [30]	2011	prospective, three-year cohort	106 pediatric patients	no	Most children with PCD remain healthy. After the 3-year follow-up period, approximately one third of patients develop VA. Intestinal deposits of tTG2 IgA identify children at risk for VA.
Lionetti et al. [23]	2012	A two-year follow-up study of 96 children	24 pediatric patients	no	The incidence of PCD and the proportion of children at risk for CD experiencing short-term decline in CD-related antibodies are substantial. A significant proportion of patients diagnosed with PCD, specifically 86%, who adhered to a normal diet became seronegative. Furthermore, the incidence of overt CD was observed in only one individual within this group.
Aurichio et al. [33]	2014	Nine-year follow-up study	210 pediatric patients	no	Patients with persistently positive antibody levels did not develop mucosal damage during follow-up.
Volta et al. [6]	2016	Prospective	77 adult patients	no	According to the findings of the three-year study, the authors do not advocate for the implementation of a GFD in asymptomatic patients with PCD, due to their limited propensity to develop VA.
Imperatore et al. [56]	2017	Retrospective follow-up study	56 adult patients	yes	Adult patients should start GFD even if not symptomatic, because of the increased risk of developing VA.
Mandile et al. [53]	2018	prospective study	47 pediatric patients	no	Only half of the patients with PCD following a GFD showed a complete clinical response. No significant differences were observed in terms of Marsh grade during the follow-up biopsy. Caution is necessary before attributing all symptoms to gluten in this condition.
Lionetti et al. [32]	2019	10 years of follow-up in a cohort of children genetically predisposed to CD.	23 pediatric patients	no	The probability of progression to overt CD while on a gluten-containing diet is extremely low in children who have been diagnosed with PCD.
Trovato et al. [21]	2019	Review	-	yes	The presence of symptoms should be considered as the main determinant to prescribe a GFD in PCD.
Newton et al. [43]	2023	retrospective	84 adult patients	yes	While PCD and CD manifest differently, the presence of non-atrophic enteropathy does not always translate into mild symptoms. The GFD proved to be effective in alleviating symptomatic PCD patients. However, VA occurred in one third of the patients who continued to consume gluten.

## Data Availability

No new data were created or analyzed in this study. Data sharing is not applicable to this article.
